# Implementing liquid biopsies into clinical decision making for cancer immunotherapy

**DOI:** 10.18632/oncotarget.17397

**Published:** 2017-04-24

**Authors:** Dagmar Quandt, Hans Dieter Zucht, Arno Amann, Anne Wulf-Goldenberg, Carl Borrebaeck, Michael Cannarile, Diether Lambrechts, Herbert Oberacher, James Garrett, Tapan Nayak, Michael Kazinski, Charles Massie, Heidi Schwarzenbach, Michele Maio, Robert Prins, Björn Wendik, Richard Hockett, Daniel Enderle, Mikkel Noerholm, Hans Hendriks, Heinz Zwierzina, Barbara Seliger

**Affiliations:** ^1^ Institute of Medical Immunology, Martin Luther University Halle-Wittenberg, Halle, Germany; ^2^ Protagen, Dortmund, Germany; ^3^ Department of Haematology and Oncology, Medical University Innsbruck, Innsbruck, Austria; ^4^ Experimental Pharmacology and Oncology, Berlin-Buch GmbH, Berlin, Germany; ^5^ Create Health Translational Cancer Center, Lund University, Lund, Sweden; ^6^ Roche Pharmaceutical Research and Early Development, Roche Innovation Center, Munich, Germany; ^7^ Laboratory for Translational Genetics, VIB Center for Cancer Biology, Leuven, Belgium; ^8^ Institute of Legal Medicine and Core Facility Metabolomics, Medical University of Innsbruck, Innsbruck, Austria; ^9^ Global Drug Development, Novartis Institutes for BioMedical Research, Cambridge, Massachusetts, USA; ^10^ Roche Pharmaceutical Research and Early Development, Roche Innovation Center Basel, Basel, Switzerland; ^11^ Qiagen, Hilden, Germany; ^12^ CRUK Cambridge Institute, Cambridge, UK; ^13^ University Medical Center Hamburg-Eppendorf, Department of Tumor Biology Hamburg, Hamburg, Germany; ^14^ Medical Oncology and Immunotherapy, University Hospital of Siena, Siena, Italy; ^15^ Department of Neurosurgery, David Geffen School of Medicine at UCLA, Los Angeles, California, USA; ^16^ Perkin Elmer, Hamburg, Germany; ^17^ Biodesix, Boulder, Colorado, USA; ^18^ Exosome Diagnostics GmbH, Martinsried, Germany; ^19^ Hendriks Pharmaceutical Consulting, Purmerend, The Netherlands

**Keywords:** liquid biopsy, biomarker, tumor, high throughput analysis, immunotherapy

## Abstract

During the last decade, novel immunotherapeutic strategies, in particular antibodies directed against immune checkpoint inhibitors, have revolutionized the treatment of different malignancies leading to an improved survival of patients. Identification of immune-related biomarkers for diagnosis, prognosis, monitoring of immune responses and selection of patients for specific cancer immunotherapies is urgently required and therefore areas of intensive research. Easily accessible samples in particular liquid biopsies (body fluids), such as blood, saliva or urine, are preferred for serial tumor biopsies.

Although monitoring of immune and tumor responses prior, during and post immunotherapy has led to significant advances of patients’ outcome, valid and stable prognostic biomarkers are still missing. This might be due to the limited capacity of the technologies employed, reproducibility of results as well as assay stability and validation of results. Therefore solid approaches to assess immune regulation and modulation as well as to follow up the nature of the tumor in liquid biopsies are urgently required to discover valuable and relevant biomarkers including sample preparation, timing of the collection and the type of liquid samples. This article summarizes our knowledge of the well-known liquid material in a new context as liquid biopsy and focuses on collection and assay requirements for the analysis and the technical developments that allow the implementation of different high-throughput assays to detect alterations at the genetic and immunologic level, which could be used for monitoring treatment efficiency, acquired therapy resistance mechanisms and the prognostic value of the liquid biopsies.

## INTRODUCTION

During the last years immunotherapies of cancer particularly immune checkpoint blockade inhibitors and adoptively transferred T cells have improved the outcome of tumor patients. Since the monitoring of tumor samples and immune responses prior and following these therapies are essential for the optimization of the mode of immunotherapy and for the selection of patients suitable for a specific immunotherapy the identification and systematic assessment of biomarkers has become an important issue for immunotherapy of cancer. In principle, liquid biopsies represent suitable samples not only for early detection, monitoring residual diseases, but also for monitoring immunotherapy responses and development of resistances, because multiple samples can be easily obtained prior, during and after immune and other therapies [1].

Liquid biopsies as a source for tumor-derived information can be analyzed at the cellular, DNA, RNA, epigenetic, protein and metabolome levels. With the development and implementation of high-throughput technologies personalized targeted and/or tumor immunotherapies have become feasible. In the future, an individual antibody binding pattern of patients might be also used for the development of personalized immunotherapy and for the monitoring of immune responses. The ultimate goal to predict immunotherapeutic success is the identification of reliable prognostic and predictive biomarkers, which link immunity with patients’ outcome thereby allowing the selection of a group of patients, who will most likely benefit from a respective treatment regimen.

The NIH/WHO international program on chemical safety has defined a biomarker as “any substance, structure, or process that can be measured in the body or its products and that can influence or predict the incidence of outcome or disease” [2].

### What will be used/monitored?

**Figure 1:**
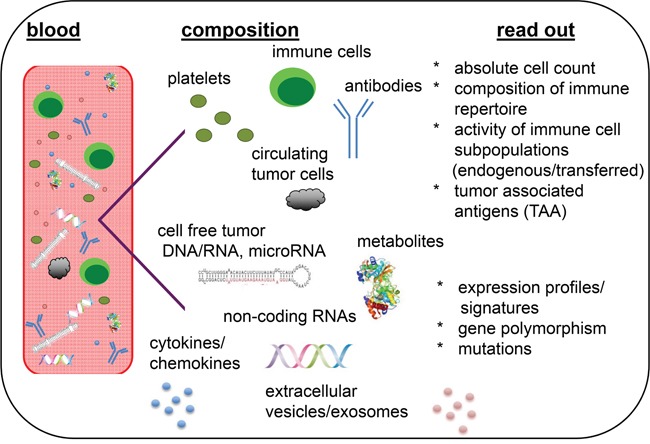
Liquid biopsies as representatives of the molecular heterogeneity and immunologic phenotype of tumors Blood serves here as representative liquid biopsy containing the highest variety on biological analytes.

For monitoring immune cell responses with long term follow-up, body fluids, such as e.g. peripheral blood and serum/plasma, should be collected at different time points prior, during and after treatment until disease progression. In addition to conventional analysis of serum markers, the absolute lymphocyte count (ALC), subtypes of lymphocytes, composition of the immune cell repertoire, activity of different immune cell subpopulations, exosome and extracellular microvesicle (EV) profiles, expression of genes/microRNAs/proteins, cytokines, metabolites, chemokines, putative tumor-associated antigens (TAA), polymorphisms in Fc receptors, antibodies and the proliferative capacity of adoptively transferred immune cells could be also analyzed in primary tumors and/or body fluids (Figure 1) [3–9].

The integration of multiple high-throughput “omics” technologies including cancer genome sequencing studies that have catalogued the mutational landscape of human tumors (e.g. single nucleotide variants, copy-number alterations, somatic events), transcriptomics for analysis of peripheral blood mononuclear cells (PBMCs) and DNA/RNA analysis in circulating tumor cells (CTC) has helped to define biomarkers. Furthermore, different proteome-based technologies, such as the serologic evaluation of proteins and antibodies (PROTEOMEX), “bottom up” and “top down” proteomics, multi-parameter ELISAs, bead or chip based affinity proteomics and multispectral imaging have been implemented for diagnosis, immune monitoring, immune response assays and/or identification of novel (immuno) therapeutic targets. However, these different profiling methods have some advantages and disadvantages (Table 1), which significantly depend on the sample preparation and handling.

**Table 1:** Advantages and disadvantages of technologies used for preclinical biomarker identification and for liquid biopsies

techniques	advantages	disadvantages
Blood count	Quick and reproducible, cheap	Limited information
Flow cytometry	Numbers, frequency and activity of specific immune cell subpopulations	Restricted to max. 30 parameters determined in parallel, data analysis requires strong expertise
NGS	High throughput analysis, identification of mutations, polymorphisms, expression profiles	Large data set, strong bioinformatics required, expensive
mRNA andmiRNA arrays	High throughput analysis, mRNA and miRNA expression pattern	Large data sets, targets not always identified
Proteomics	High throughput analysis, protein expression pattern	Large data set, strong bioinformatics required, depending on the method very challenging
IHC	Frequency and localization, comparison to liquid biopsy data	Not always antibodies available for paraffin-embedded tissues
MSI	Localisation and distance of immune cells	Not an established procedure for different immune markers
Metabolomics	High through put analysis	Large data sets, bioinformatics required, high dependence on patients life style
Luminex/ELISA	Stable, reproducible assay	Limited information due to limited parallel analytes

Early biomarker screening prior and during therapy could help to timely identify non-responders, and accordingly discontinue time- and cost-sensitive therapies in order to give patients the chance to switch to alternative treatment regimens. Such screening is mainly based on the identification of a biomarker signature rather than one particular biomarker, since a single biomarker cannot encompass all informations needed. A set of distinct biomarkers should be able to (i) discriminate between different tumor entities rather than merely between tumor patients and healthy individuals, (ii) discriminate different (early and advanced) tumor stages or subtypes and (iii) guide selection of efficient treatment options.

### What is currently discussed to serve as (immune) biomarker in the blood?

Cell-free nucleic acids (cfNA) including DNA, RNA and Ago2-bound microRNAs (miRNAs), as well as CTC, exosomes and other EVs, protein panels, (auto)-antibodies, diverse metabolites, soluble mediators and changes in the frequency, composition and/or activity of (immune) cell populations are currently discussed as minimally-invasive biomarkers, which are easily accessible in blood samples from tumor patients to detect premalignant and early-stage cancers, to monitor response to treatment and development of therapy resistance [9–16].

Recently, exosomes have gained interest to advance biomarker research, since these nanometer-scale vesicles are secreted by living cells and carry the full range of molecules, e.g. DNA, RNA, miRNAs, proteins, cytokines and chemokines from their originating cells [6]. Due to their encapsulation in the bi-lipid membrane envelope their cargo is protected from enzymatic degradation and thus possesses a high biomarker potential [17]. Different methods [19] allowing the purification of high quality exosomal RNA (ExoLution™) or the simultaneous co-isolation of both cell-free DNA and exosomal RNA (ExoLution™ Plus) have been recently developed [20], which result in the isolation of EVs independent of their cellular origin. While almost all cell types secrete such vesicles, large number of vesicles will be also obtained from normal, non-cancerous cells, a problem, which has to be solved by the identification of cancer-derived exosomes as recently suggested for pancreatic cancer patients [21].

Circulating cell-free and exosomal DNA and RNA in human blood is derived from different cellular sources, including blood and tumor cells. It is postulated that circulating tumor DNA (ctDNA) reflects the molecular changes occurring at the tumor site in real time [22]. Indeed, the identification of somatic mutations within the epidermal growth factor receptor (EGF-R) gene using ctDNA isolated from plasma with the *therascreen* EGF-R Plasma RGQ PCR Kit [23] was successful. The kit was originally developed for tissue biopsies and was successfully implemented for non-small cell lung carcinoma (NSCLC) patients. The EGF-R mutational analyses of tissue biopsies provided evidence for predicting patients that respond to and clinically benefit from treatment with afatinib, erlotinib or gefitinib [24] and can also be applied for analyses of EGF-R mutations in blood samples [23]. CtDNA analysis is able to give a global view of cancer genomes across tumor sites [24–26] and can be used to monitor therapy response [27]. Due to the short half-life of ctDNA (± 2 hrs) and early changes following cytotoxic treatment ctDNA may provide an indication of tumor response [30, 31]. Therefore ctDNA analysis may prove to be useful for molecular stratification (e.g. exome-seq for mutation load or neo-antigen prediction), monitoring response (e.g. individualized TAA-seq for early kinetics and relapse) and for molecular profiling of relapsing patients (e.g. exome-seq to study tumor evolution) undergoing immunotherapy. Due to the chromosomal instability of tumors, analysis of plasma ctDNA is a suitable tool for determination of copy number profiles in comparison to patients’ biopsies [32]. Analogous to exosomes also cfNA might be tracked back to their origin (e.g. tumor, immune or cells of other origin) as recently investigated in plasma samples derived from gastric cancer patients using MYC and HER2/neu genes known to be amplified in this cancer type [29, 30].

Circulating miRNAs in serum or plasma might be also used as biomarkers due to their high stability under storage, easy handling conditions and emerging expression signatures that are associated with cancer survival and therapy response [35]. Plasma/serum miRNAs exist as cell-free, protein-bound molecules that are released by apoptotic and necrotic cells into the blood circulation, but are also actively released in exosomes. In HER-2/neu-positive breast cancer patients, high serum levels of cell-free miR-21 correlate with a shorter overall survival prior and after neoadjuvant therapy [32]. In ovarian cancer patients elevated serum concentrations of cell-free miR-429 are associated with advanced FIGO stages, higher values of the tumor marker CA125 and a poor overall survival rate [33]. In breast cancer patients the serum levels of exosomal miR-373 are significantly higher than those of cell-free miR-373 and associated with estrogen-negative receptor status and apoptosis [34]. It is still discussed whether the majority of circulating miRNAs is packaged into exosomes [39] or rather exists as Ago2-bound complexes [36], but both populations can be readily detected in plasma [20].

Although more than 50 years ago CTCs in the periphery of solid tumor patients have been identified, CTCs are still under critical review concerning their usefulness as biomarkers in cancer patients [41]. The biggest challenge is the low number of 1-10 CTCs/ml of blood thereby making their analysis both in terms of sensitivity and specificity very difficult.

Single or panels of proteins (≥ 10 analytes) from serum that address soluble immune-regulatory molecules, such as cytokines, chemokines and co-stimulators [38], acute phase proteins, complement and innate mediators as well as growth/apoptosis markers have been proven as biomarkers for the stratification of patients. These markers can be determined by multiplex analysis e.g. Luminex.

Potentially of high importance are the absolute numbers and relative proportions of immune cell populations within the patients’ tumor and immune cell populations, in particular in blood, lymph nodes and the bone marrow. The significance of the immune cell composition, the frequency as well as the localization of immune cell subpopulations within the tumor quantified in the “Immunoscore” [43] has been established in the last years and might be at least for colorectal cancer (CRC) superior than TNM grading and staging. However, since it requires tumor tissues and high skilled manpower alternative measurements with similar power are currently developed, such as multiplexing with centralized digital pathology (multispectral imaging) and/or multiplex tumor flow cytometry. Features of immune cells in terms of their differentiation and activation status as well as their effector potential are currently analyzed, in particular in blood of tumor patients undergoing diverse immunotherapies. This led for example to the identification of the prognostic value of peripheral blood CD8^high^CD57^+^ lymphocytes regarding recurrence-free survival of patients with non-muscle-invasive bladder carcinoma after transurethral resection and intra-vesical IL-2 treatment [40]. Other studies suggest that quantities and proportions of T (mainly CD8^+^) lymphocyte subsets in PBMCs are related to the increased survival of cancer patients already serving as biomarkers in myeloma [45]. Functional analyses for CD8^+^ T cell cytokine responsiveness as measured by changes in the phosphorylation status of STAT molecules might serve as biomarker discriminating glioblastoma patients benefiting from a DC vaccine [46].

Several studies imply that cancers with higher mutation burdens, such as microsatellite instability associated with a greater likelihood of expressing neo-antigens, are most likely to respond to checkpoint blockade inhibitors [7]. However, a broader picture of the patients’ tumor mutational load along with the status of the immune system as shown for the therapeutic success of anti-CTLA-4 (ipilimumab) [43] is needed to predict a patients’ response to immunotherapy [44]. So far, this information was obtained by the use of difficult to access and repeated sampling from tumor tissues. This type of analyses is currently translated to liquid biopsies using the potential of circulating immune cells, ctDNA, CTCs, or exosomal RNA to identify patients’ overall tumoral mutation load or specific TAAs from blood. Additional, analyses of protein levels for immune markers would increase the knowledge of the patients’ blood immune contexture.

While the induction of immune-related adverse events (irAEs) is a serious concern of immune-checkpoint blockade inhibition observing an inflamed tumor environment as well as increased preexisting autoantibodies, parameters that might act as markers of immune responsiveness bear the potential as clinical biomarkers [45, 46]. Thus monitoring auto-reactivity and anti-tumor reactivity by screening of antibodies should be included in a comprehensive biomarker strategy.

### Sample preparation and difficulties in harmonizing biomarker detection

Sample preparation and generation is an important issue, since significant differences have been observed between distinct methods and consumables used for serum, plasma and immune cells obtained from PBMCs. In detail, the standby times until centrifugation (1 – 48 hrs), the blood collection method, nucleic acid preparation methods, exosome purification methods, methodical aspects for flow cytometry and the IgG purification from these samples could affect the analysis and result in reproducibility problems. Furthermore, if immune parameters are monitored, information about chemotherapy, anti-inflammatory drugs and hormone therapy is crucial, since these will most definitely affect the immune signatures. International consortia are currently establishing standard practices for blood collection and processing for circulating biomarkers (e.g. SPEDIA [51] and CANCER-ID [52]).

Other often neglected preclinical variables are the condition of the patients at the time of the specimen collection and by the specimen collection process itself. Common examples of patients’ condition include the duration of a fasting state/diet for blood lipids, glucose and other parameters. In addition, the time of day for blood collection affects cortisol and albumin levels [53]. The challenges with blood as for any other material undergoing biomarker analyses are the differences in the routine handling procedure, assay performance and inter-individual variability.

Two distinct strategies to synchronize biomarker detection are currently discussed in the literature: (i) the standardization of assays and (ii) the harmonization of assays. The first requires the exact same assay with the same standard operating procedures (SOP) and well-trained personal is needed in all laboratories, which is hard in terms of costs and feasibility. The second, more accepted way of handling this issue is the harmonization that requires trained personal and standardized laboratory equipment, not the exact same assay kits, but positive quality controls that have to be implemented in every assay in all cooperating/participating laboratories and research centers [9]. Successful harmonization of assays has been achieved exemplarily for the enzyme-linked immune spot (ELISPOT) technology aimed in recognizing antigen specific T cells in blood samples. After the stepwise introduction of harmonizing workflow guidelines the success rate of the participating centers in detection of rare antigen specific T cells within the central distributed standardized quality control samples increased dramatically from 53% to 93% [50].

### Preclinical biomarker identification

Biomarker development following a traditional stepwise process from cell culture via mouse models to patient's samples has helped, but also often failed in generating novel biomarkers with clinical relevance [55]. To identify new/improved biomarkers different well-known, but also new experimental approaches have to be implemented. Therefore, techniques and strategies like 3D culture, humanized mouse models and plasma protein profiling using recombinant antibody microarrays are discussed in this section [56, 57].

### 3D models *in vitro*

To explore cell biology and drug efficacy, cell-based assays were employed, in which tumor cells were typically grown on two-dimensional plastic surfaces or as single cell suspensions. However, since cell biology is profoundly influenced by its microenvironment, cell-based assays should be rather performed using 3D cultures, since they reflect both extracellular matrix and cell-cell contacts, such as cell-matrix interactions. These techniques were successfully implemented to study tumor-stroma interactions in NSCLC or tumor immune cell infiltration [51, 52]. This innovative method has the potential to improve the search for new immune biomarkers that could predict the patients’ responsiveness to immunotherapy. 3D culture models could be used to test the patients’ antitumor immune system and its modulation by immunotherapies in an *ex vivo* tumor micro tissue. A potential biomarker in this sense would have the capacity of a particular immune cell type to enter the tumor micro tissue or to change the effector function inside the tumor micro tissue upon a given immunotherapeutic intervention.

### Experimental animal models

Very old, but still valid tools to study human diseases are experimental mouse models. Initially murine syngeneic mouse models or immune deficient human xenograft models were used to study new (immuno) therapeutic strategies to treat cancer. These models have major drawbacks since the tumors are either not of human origin or are human or even patient derived xenografts (PDX), but lack the immune system and the human stroma environment. In particular immunotherapies, such as checkpoint inhibitors like anti-PD1/anti-PD-L1 cannot be tested in immune deficient mice, since their intervention relies on the existing immune system. To circumvent these problems humanized mouse models have been established, in which the murine immune system is replaced by the human immune system using PBMCs or bone marrow stem cell transplantations [60]. These humanized mouse models are still under development, since they are difficult to handle and they do not reconstitute a complete human immune system [60]. However, in such humanized mice PDX can be established, which allow to study any of the new (immuno) therapeutic strategies in preclinical *in vivo* immunocompetent models over time. Thus, such model systems have the potential to lead to the identification of blood-borne biomarkers that predict patients’ responsiveness to immunotherapy.

### Proteome/metabolome-based technologies

Other technologies are proteome-based, such as e.g. the Global Proteome Survey GPS that employs an oriented single chain antibody fragment immobilization approach with known antibodies and with context-independent motif specific (CIMS) antibodies that recognize peptides of hundreds of proteins allowing for a target discovery [61]. One big advantage of this technology is the low amount of blood in the μl range required for analysis. Using this technology, breast cancer has been graded with a significantly higher resolution when compared to conventional pathology [55]. Furthermore, using recombinant antibody microarrays serum protein signatures of immune regulatory molecules (cytokines, chemokines and co-stimulators) and unidentified peptides have been identified in a multicenter trial for pancreatic ductal adenocarcinoma, which allowed discriminating cancer patients from controls [38, 56]. Even more importantly, a large validation set of over 1300 pancreatic carcinoma patients and controls from two centers in Denmark identified a similar serum signature, which discriminated non-cancer patients from stage I and II pancreatic cancer patients. These data are of high significance for a potential early cancer diagnostic kit, resulting in a dramatically increase in patients’ survival chance for this particular cancer type normally displaying a dismal outcome with 5 year survival of 5-7 %. However, if early stage I/II disease can be detected the 5-year survival could increase to over 50 % (Japanese Pancreas Society). Currently, prospective studies are on the way in the USA. This demonstrates the potential in precision diagnostics [63].

A very interesting additional family of analytes with the potency to serve as biomarkers in blood samples are metabolites [64] including immune modulating amino acids, (anti)-oxidants and members of different lipid classes. In a study with breast cancer patients receiving endocrine therapy with tamoxifen several metabolites from different classes were altered in patients under tamoxifen when compared to cancer patients not receiving this treatment.

### Auto-antibody detection

Furthermore, the use of auto-antibodies as biomarkers was exemplified in dissecting prostate cancer patients using a combination of different techniques such as protein arrays and Luminex that are spiked with auto-antigens (4012 or 3061). These results were validated on tissue microarrays (TMAs) and by qPCR of tissue samples. A different auto-antibody panel comprised of 165 antibodies (Ab) was found in serum of high when compared to low inflammation prostate cancer patients [59]. Of note, among the respective auto-antigens only Spastin (SPAST) was significantly increased in high versus low inflammation patients, while mRNA levels for all auto-antigens were unaltered. Thus no direct correlations between antibody titers in serum and their protein/mRNA expression levels exist in prostate cancer.

### *In vitro* diagnostic (IVD)

IVD development in companies for biomarker identification is time consuming due to an inadequate understanding of the modes of action of therapeutic targets, inadequate preclinical models for discovery and validation in addition to the separation of the noise from real data and the cross-validation of a specific marker in an independent large cohort of patients. Another big challenge is the broad diversity in the patients’ cohort due to inter- and intra-heterogeneity of a particular disease [66–68]. One or a panel of identified biomarker(s) might not hold true for a complete patients’ cohort giving the need for individual consideration and the definition of inclusion/exclusion criteria. To figure this out, a close collaboration between physicians, academic research institutes and pharmaceutical industry partners is needed.

### Liquid biopsies from cerebrospinal fluid, saliva, urine and other sources

In addition to blood as the most abundant liquid body fluid there are other body liquids to study biomarkers, such as ascites/pleural fluid, cerebrospinal fluid (CSF), bronchial alveolar lavage (BAL), urine and saliva/sputum (Table 2). Among these alternative liquids, saliva and urine are non-invasive and therefore of high interest to clinicians as current and future biomarker source. Collecting body fluids proximal to the tumor site of interest can significantly increase sensitivity for detection and monitoring [60]. Common to all alternative liquids for biomarker analysis and different to blood is the appearance of immune cells only in the case of inflammation or presumably late stage organ damage. All other (ctDNA, exosomes, proteins, metabolites and antibodies) mentioned blood biomarker will be testable to varying degrees in these alternative liquids.

**Table 2:** Non-invasive and invasive body fluids for the identification of tumor-derived information

body fluids	non-invasive	Invasive
Peripheral blood	✓	
Serum/plasma	✓	
Saliva	✓	
CSF		✓
Urine	✓	
BAL		✓
Pleural effusion		✓

Compared to blood saliva contains a similar variety of DNA, RNA, proteins, metabolites, and microbiota that can be compiled into a multiplex of cancer detection markers [61]. Urine has already been a well-studied source for biomarker analysis in different cancer types, such as, bladder cancer [62], kidney cancer [63], ovarian cancer [63] and prostate cancer [74]. Recently, urine samples were used in a study of more than 1500 patients of prostate cancer to predict high-grade prostate cancer by analysis of RNA from urinary exosomes [65]. Ascites has been analyzed in ovarian cancer patients for biomarkers associated with disease prognosis and patients’ outcome. In these patients high IFN-γ levels correlated with an increased overall survival [66]. This list could be extended to multiple other references on different liquid samples, which is not the prime focus of this article.

### Individualized diagnostic and therapy

The most cost and effort effective way would certainly be to use standardized assays for biomarker analysis for a large number of patients. Since one faces a high patients’ cohort diversity, these standardized assays for a particular type of cancer disease might not be applicable. The profiling of blood samples might allow reducing the number of patients’ subgroups that require analyses of tissue biopsies [77, 78]. However, these patients’ subgroups would not allow predicting the clinical performance of the given patients’ group according to the current paradigm of “personalized medicine”. Thus, the logical extension of personalized medicine would tailor treatment to the specific individual without prior subgrouping of patients.

Novel individual therapeutic options include the alteration of the epigenetic features of tumors, in particular histone modifications and DNA methylation. Interestingly, the methylation status of melanoma patients does correlate with the overall survival [67]. Furthermore, *in vitro* data and *in vivo* preclinical mice data show re-expression of cancer testis antigens, improved anti-tumor immunity and better tumor control upon treatment with hypomethylating agents and/or histone deacetylation inhibitors. These data provide the rational for the use of hypomethylating agents and histone deacetylation inhibitors in melanoma patients. In the future, the methylation status should be investigated in liquid biopsies prior to individual therapy decision.

Properly controlled tissue acquisition and multiplex immunohistochemical analysis might overcome issues related to pre-analytical and analytical variability leading to a more reproducible assessment of clinically meaningful biomarkers. However, this technology still has to be improved and optimized due to the limited tissue material available in the clinical setting. The analysis of multiple immune parameters in tissue biopsies by multispectral fluorescent immunohistochemistry is an important advancement and has recently been successfully implemented in some clinical studies [68, 69]. This technique has several advantages, since it allows (i) a combination of different markers on a single histology section, (ii) the calculation of distances between individual cell types and (iii) the usage of already established antibodies for immunohistochemistry by only adding an additional labeling step [82].

### Other non-invasive methods to image therapy success

The success rate of cancer immunotherapy is difficult to predict, as its efficacy often depends not only upon characteristics of the tumor lesions, but also of the tumor microenvironment involving immune cells and soluble mediators. Molecular and functional imaging with Positron Emission Tomography (PET) and Magnetic Resonance Imaging (MRI) allows repeated non-invasive *in vivo* measurement of many critical molecular features of tumor lesions and microenvironment, such as metabolism, proliferation, hypoxia, cell death and immune cell infiltrate, which can assist the knowledge of how cancer immunotherapy works and also facilitates clinical decision making [84].

More conventional PET imaging tracers such as ^18^F-FLT and ^18^F-FDG-PET were successfully used to study the kinetics and involvement of lymphocyte subsets in response to vaccination. These tracers incorporate in highly proliferative cells and are therefore not specific to cell types, but when locally applied e.g. in the tumor draining lymph node in conjunction with a DC vaccine, they reflect lymphocyte proliferation to vaccination. This technique allows an early discrimination between responding and non-responding patients in anti-cancer vaccination protocols and aids physicians in individualized decision-making processes [84].

New imaging tracers specifically targeting subsets of immune cells by the use of radiolabeled fragmented specific antibodies to CD8^+^ and CD4^+^ T cells (use of cys-diabody and minibody) and CD11b^+^ myeloid cells (use of variable domain of a camelid heavy-chain antibody) are successfully used in preclinical mouse models and currently being developed for advanced PET imaging in clinical monitoring applications to cancer immunotherapy [85–87].

### From biopsy to peripheral blood

One of the major challenges remains to establish the biological connection of peripheral biomarkers and the complex tumor immune contexture. There is still a debate of whether peripheral immune cells are representative for the anti-tumor immunity observed in the tumor microenvironment (TME). Particularly, the link between peripheral blood immune cells and the prognostic and predictive relevance of the spatial distribution of immune cells in the tumor tissue is still unclear [76]. This is related to the fact that activated immune cells detected in the blood, primary or secondary lymphoid organs may be silenced locally by tumor cells or by immune suppressive cells e.g. regulatory T cells (Treg), myeloid derived suppressor cells (MDSC), tumor-associated macrophages (TAM) or cancer associated fibroblast [76]. Further, it is still not fully understood whether cancer cells release an equal amount of CTC, exosomes or ctDNA during disease progression and in response to treatment with a given therapy into the periphery. Overall, more interventional studies with integrated blood and tissue biomarkers combining proteomic and genomic multiplex approaches are needed to identify the clinical relevance of blood-based markers. However, due the invasive nature of tumor biopsies and the limitation to obtain multiple biopsies for serial assessments in the clinical routine, further investigation of blood based markers as a relevant surrogate tissue is needed.

The cellular sources of blood not only include the “classical” immune cells, but also CTCs of diverse tumor entities, hematopoietic stem cells, erythrocytes and platelets. In hemostasis, platelets have been demonstrated to be more present than effector cells and represent the major inflammatory cells mediating inflammasome signaling thereby affecting both innate and adaptive immune responses [89]. This led to the assumption that platelets are involved in the development of cancer and in success/failure of cancer therapies, which is a rather new and interesting field for basic researchers and clinicians [78].

### Controversial discussion of tissue PD-L1 expression as biomarker: alternatives may predict treatment success to PD-1/PD-L1 axis using liquid biopsies

Recently, the use of antibodies directed against different checkpoint targets, such as anti-PD-1 (Nivolumab, Pembrolizumab and Pidilizumab), anti-PD-L1 (MS-936559, MEDI4736, and MPDL3280A) and anti-CTLA4 (Ipilimumab) has been successfully implemented for the treatment of a number of different tumor entities [79]. For anti-PD1/-PD-L1 treatment, the immunohistochemical analysis of PD-L1 expression on the surface of tumor cells was suggested as a suitable biomarker to predict therapy success. It was postulated that blocking PD1/PD-L1 could be used in patients’ with tumors expressing PD-L1. PD-L1 positive tumors are known to cause impaired T cell effector function by binding to PD-1 on T cells. However, the use of PD-L1 as biomarker for melanoma and lung cancer [80, 81] is still under debate. This is due to conflicting results in different patients’ cohorts by lack of standardized methodology, lack of consensus on the “best” antibody associated with a lack of standardized cut off values for sample positivity or values of intensity and lack of agreement on cells to analyze. Additionally, PD-L1 and PD1 are not exclusively expressed on tumor tissues or effector T cells, but are rather widely distributed among immune cells and other tissue cells, present in the TME. Furthermore, it is noteworthy that at least one known additional ligand for PD-1, the PD-L2, also known as B7-DC, exists, which is not blocked by anti-PD-L1 antibodies [94]. Even more complexity arises from the fact that the existence of soluble forms of PD-L1 has been confirmed, which might affect the efficacy of an anti-PD-L1-based therapy [83, 84].

Alternatively to PD-L1 expression on tumor cells as a biomarker, one study found a significant correlation of high tumor PD-L1 expression on immune cells infiltrating the tumor as a predictive marker for therapy success [85]. The success of anti-PD-1 therapy appears to rely on the pre-existence of intra-tumoral CD8^+^ T cells with proliferative capacity [86, 87]. Other factors, such as increased density and decreased diversity in antigen specificity of T cells within the tumor, may also provide predictive value, as observed in melanoma patients treated with PD-1 mAb [87]. However, these alternative profiling strategies have not yet been validated on different cohorts or/and tumor entities. This is in line with a number of other putative novel published biomarkers, which often has either not yet been validated or the validation attempts from other laboratories with another patients’ cohort failed. However, a multi-parameter serum analysis demonstrated differentially expressed analytes in responder and non-responder patients upon nivolumab treatment in melanoma patients (personal communication R. Hockett). These promising data have already been validated in a second patient cohort and are currently analyzed in a prospective study.

### Pros and cons of blood biopsies

**Figure 2:**
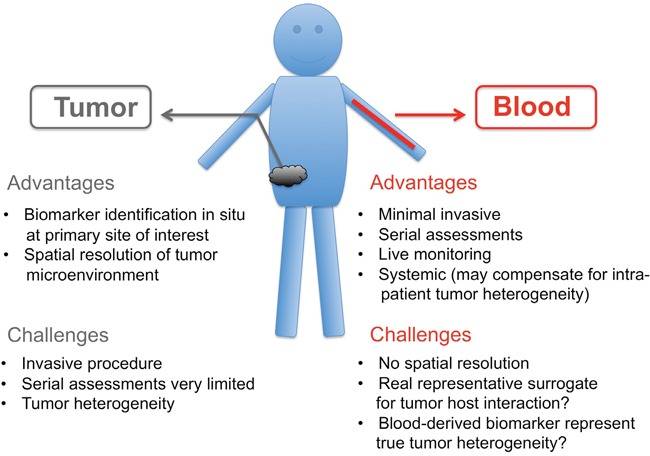
“Pros” and “Cons” of tumor biopsies versus blood biopsies

There exist a number of “pros” and “cons” in terms of biomarker analysis for both blood samples and the current gold standard, tumor biopsies. The advantages of tumor biopsies are (i) the *in situ* analysis of the primary site of interest as well as (ii) the spatial resolution of the TME known as the tumor landscape. Disadvantages of tissue biopsies are (i) the invasiveness of the method, (ii) lack of willingness of patients for the necessary surgery, (iii) serial assessment and of high importance, (iv) the tumor heterogeneity (intra and primary versus metastases). The advantage of blood samples are (i) minimal invasive, (ii) serial assessment, (iii) live monitoring and (iv) systemic view that may account of tumor heterogeneity (v) the speed and reduced cost of sample preparation and (vi) the easy access of blood material from healthy volunteers or patients’ with different diseases for comparison. The disadvantages on the other hand are (i) no spatial resolution of tumor microenvironment (ii) lack of knowledge whether the blood reflects the “true” host tumor interaction and (iii) uncertainty whether the blood sample overcomes the tumor heterogeneity issue (Figure 2). However, clinical data to address some of these concerns are starting to accumulate, and some ctDNA-based assays have already been found to be as predictive as tissue in recent retrospective clinical studies [88]. As a definitive sign that liquid biopsies are starting to enter clinical practice several liquid biopsy IVD products are already available, e.g. FDA recently approved Roche cobas EGF-R mutation test for detection of EGF-R mutations in plasma of NSCLC patients as a Companion Diagnostic test to Tarceva [101].

More broadly, a comprehensive and integrated bio-sampling approach of tissues and PBMCs in the clinical setting and preclinical models may allow the identification of less invasive biomarkers that reflect clinically meaningful aspects of the immune tumor microenvironment, improving the triage and management of patients in cancer immunotherapy. Overall, new tools for tissue analysis, rigorous validation and standardization of methods will help to better understand the dynamic nature of immune-tumor interaction.

## CONCLUSIONS

The clinical implementation of blood-derived biomarkers to predict tumor progression, response to therapy, therapy resistance or even early diagnosis of tumor occurrence is certainly wanted and urgently required. Recent progress is promising and the identification of the “right” biomarker/biomarker panel to define different tumor entities, if possible at distinct disease stages, is emerging, but needs to overcome current challenges of sample preparation, standardization of techniques, and acceptance in clinical practice. Even though, prospective studies that validate the liquid biopsy data in independent patient cohorts are crucial and are still missing in most cases, the so far obtained biomarkers from retrospective studies using liquid biopsies are very encouraging Despite these advances there is still an urgent need of blood analysis as a potential surrogate for tissues samples as summarized in Figure 2. Some of the different biomarkers presented here might serve as diagnostic tools, such as tumor specific auto-antibodies, which are long lasting. Others might be implemented for real-time monitoring of therapy success, such as DNA and RNA mutations, since these alterations are reflecting disease burden. Blood- or tumor-derived miRNAs seem to illuminate the topic of therapy responder/non-responder and also could timely uncover therapy success. Protein biomarker panels have shown accurate diagnostic abilities, although further research is needed to identify their true nature in the context of different cancer indications. Here, exosomes may allow biofluid-based detection of membrane surface proteins from the tumor microenvironment. Liquid biopsies are a powerful tool that fosters efficient new (immuno) therapeutic implementations to treat cancer and have the potential to significantly reduce tumor biopsies.

## Abbreviations

Ab: antibody
ALC: absolute lymphocyte count
BAL: broncho alveolar lavage
cfNA: cell-free nucleic acid
CIMS: context independent motif specific
CRC: colorectal cancer
CSF: cerebrospinal fluid
ctDNA: circulating tumor DNA
CTC: circulating tumor cells
EGF-R: epidermal growth factor receptor
EV: extracellular vesicle
miRNA: microRNA
MSI: multispectral imaging
NSCLC: non-small cell lung carcinoma
PBMC: peripheral blood mononuclear cells
PDX: patients’ derived xenografts
SNP: single nucleotide polymorphisms
SOP: standard operation procedures
TAA: tumor associated antigen
TMA: tissue micro array
TME: tumor microenvironment
